# Oxidative Stress in *Lactuca sativa* L.: Cellular Mechanisms, Stress Responses, and Mitigation Strategies

**DOI:** 10.3390/plants15193069

**Published:** 2026-10-08

**Authors:** Jorge Octaviano Gomez-Castrejon, Claudia Jazmin Ramos-Torres, María Myrna Solís-Oba, Liliana Alamilla-Beltran, Hugo Martínez-Gutiérrez, María de Jesús Perea-Flores

**Affiliations:** 1 Escuela Nacional de Ciencias Biológicas, Instituto Politécnico Nacional, Unidad Profesional Adolfo López Mateos, Zacatenco, Av. Wilfrido Massieu 399, Col. Nueva Industrial Vallejo, Alcaldía Gustavo A. Madero, Ciudad de México 07738, Mexico; jogomezc1800@alumno.ipn.mx (J.O.G.-C.); lalamilla@ipn.mx (L.A.-B.); 2 Instituto Politécnico Nacional, Centro de Nanociencias y Micro y Nanotecnologías. Av. Luis Enrique Erro S/N, Unidad Profesional Adolfo López Mateos, Zacatenco, Alcaldía Gustavo A. Madero, Ciudad de Mexico 07738, Mexico; cramos@ipn.mx; 3 Centro de Investigación en Biotecnología Aplicada, Instituto Politécnico Nacional, Ex-Hacienda San Juan Molino, Carretera Estatal Tecuexcomac-Tepetitla Km 1.5, Santa Inés Tecuexcomac, Tlaxcala 90700, Mexico; msolis@ipn.mx

**Keywords:** antioxidants, oxidative stress, lettuce, nanoparticles, reactive oxygen species (ROS)

## Abstract

Lettuce is a vegetable crop of major importance in the global diet due to its low caloric content and high levels of fiber, minerals, vitamins, and antioxidant compounds. China is the world’s leading lettuce producer, accounting for more than 50% of global production. However, climate change and anthropogenic activities expose agricultural crops to both biotic and abiotic stresses, including drought, salinity, and heavy metal contamination. These adverse conditions induce the overproduction of reactive oxygen species, resulting in redox imbalance that damages lipids, proteins, and nucleic acids, ultimately leading to wilting, chlorosis, necrosis, and reductions in plant growth and nutritional value. To counteract cellular damage, this review describes various strategies for mitigating oxidative stress in lettuce crops. These approaches include the exogenous application of antioxidants and osmoprotectants, the use of biostimulants, nanopriming techniques, nanoparticles, encapsulated delivery systems, genome editing, and genetic engineering. Finally, this review concludes that oxidative stress management should evolve toward an integrated, precision-based approach combining two or more complementary strategies to ensure crop sustainability, commercial yield, and food security under challenging environmental conditions.

## 1. Introduction

Lettuce (*Lactuca sativa* L.) is a widely cultivated and consumed leafy green vegetable worldwide, characterized by its high dietary fiber content, abundance of vitamins (A, C, and K), minerals (Ca, Fe, and Mg), and natural antioxidants [1,2], as well as its low caloric content. Lettuce is produced under open-field, greenhouse, and hydroponic cultivation systems [3,4]. Owing to its versatility and ease of cultivation, lettuce has become an essential component of healthy diets and intensive agricultural systems, which are characterized by high crop density, efficient use of space, and optimization of agricultural inputs to maximize production per unit area. The global lettuce market size was valued at USD 3.86 Billion in 2024 and is poised to grow from USD 4.02 Billion in 2025 to USD 5.59 billion by 2033 [5].

The major lettuce-producing countries include China, the United States, India, Spain, Italy, and Mexico, with China being the world’s leading producer, accounting for more than 50 % of global production. This predominance has been supported by the implementation of technologies such as vertical farming and LED lighting, among others [6,7]. At the national level, Mexico has become the world’s third-largest lettuce exporter, accounting for 23.3% of global exports in 2022 [7]. National production reached 539,000 tons in 2020, compared with 516,000 tons in 2019 [8], and 512,431.40 metric tons in 2024 [9]. Romaine and Orejona lettuce are the predominant varieties produced in Mexico, together accounting for 98% of the national harvest, whereas Baby Leaf and Escarole are produced on a smaller scale [8,10]. The leading lettuce-producing states are Guanajuato, Baja California, Zacatecas and Puebla, with 98% of production exported to the United States.

In recent decades, lettuce production has shown an upward trend driven by population growth, increasing demand for fresh and healthy foods, and the development of more efficient agricultural technologies [11,12]. Cultivation systems have become diversified, with an expansion in the use of hydroponic and greenhouse systems compared with conventional open-field cultivation [10,13]. Particularly in Mexico, hydroponic lettuce production has been increasingly implemented, improving profitability and product uniformity while shortening production cycles. Systems such as the Nutrient Film Technique (NFT) and deep-water culture have demonstrated high efficacy and efficiency in terms of crop yield and resource utilization [10,14].

Optimal lettuce growth requires strictly regulated environmental parameters (Figure 1). Air temperatures between 20 °C and 24 °C have been identified as ideal for maximizing leaf expansion, shoot biomass, and water content, while minimizing physiological disorders such as tipburn and premature bolting. Root-zone temperatures around 28 °C further improve nutrient uptake and dry matter accumulation, especially under controlled light conditions [15]. Relative humidity levels between 60% and 80% favor the maintenance of turgor and reduce transpiration stress, although excessive humidity can predispose plants to fungal infections [16]. Nutritional requirements are equally critical: nitrogen (N) is essential for chlorophyll synthesis and leaf development; phosphorus (P) favors root growth and energy transfer; and potassium (K) improves stress tolerance and osmotic regulation. Micronutrients such as calcium (Ca), magnesium (Mg), and boron (B) play a role in cell wall integrity, photosynthesis, and meristem stability. In hydroponic systems, maintaining the nutrient solution temperature around 21.1 °C has been shown to increase shoot fresh weight by 15% compared with ambient conditions, and crop-specific responses influence maturity and overall quality [17].

From a nutritional perspective, lettuce is characterized by its low energy content and its significant contribution to the dietary intake of water, dietary fiber, vitamin C, and folate, as well as minerals such as potassium and magnesium (Table 1). Furthermore, lettuce is an important source of bioactive secondary metabolites with antioxidant potential. These compounds have been associated with beneficial effects on human health, including protection against oxidative stress and the modulation of inflammatory processes [19,20,21]. Recent studies have also demonstrated that the phytochemical content of lettuce can vary according to environmental factors, including light intensity and spectral quality during cultivation. These findings have stimulated research into the use of LED lighting in hydroponic systems as a strategy to enhance the nutraceutical value of this vegetable crop [22].

Agricultural crops such as lettuce are increasingly affected by changing environmental conditions, particularly those associated with climate change and anthropogenic activities. Lettuce is particularly susceptible to oxidative stress, especially under conditions of salinity, drought, heavy metal contamination, and extreme temperatures [24,25]. Oxidative stress arises from an imbalance between reactive oxygen species (ROS) production and the plant’s antioxidant capacity, thereby impairing key physiological processes such as photosynthesis, nutrient uptake, and cellular integrity [26,27]. Several strategies have been developed to counteract the detrimental effects of oxidative stress in lettuce, including the exogenous application of antioxidants such as ascorbic acid, glutathione, and β-carotene [24,28]; the use of ZnO nanoparticles to enhance tolerance to salinity and heavy metal stress [25]; and plant biotechnology and genome-editing approaches aimed at enhancing the expression of antioxidant-related genes [6].

Within this context, understanding the strategies used to mitigate oxidative stress in lettuce is particularly important given the crop’s agronomic, nutritional, and commercial relevance. Therefore, the aim of this review is to provide an integrated analysis of the major sources of oxidative stress in *Lactuca sativa* L. and the technological strategies that have demonstrated efficacy in reducing their detrimental effects. In addition, emerging approaches that could enhance stress tolerance and crop quality in current production systems are discussed. Throughout this review, evidence obtained directly in *Lactuca sativa* L. is prioritized. When lettuce specific information is unavailable, the studies conducted in general plant models are included only to support mechanisms and are explicitly identified as non-lettuce evidence. Special emphasis is placed on cadmium rather than other heavy metals because it is found at the highest concentrations in agricultural irrigation water and moves easily through soil, quickly reaching plant roots [29].

## 2. Environmental Stress

Environmental stress is defined as the pressure exerted on plants by external factors, that exceed their normal physiological tolerance range and compromise essential processes such as photosynthesis, water and nutrient uptake, secondary metabolism, and plant tissue integrity [30,31]. Environmental stress can be classified as biotic and abiotic (Figure 2) [32]. The combination of these factors, particularly under extreme conditions, amplifies the pressure on the plant and generates significant physiological and metabolic alterations.

Prolonged exposure to high temperatures accelerates bolting and reduces leaf quality by affecting photosynthesis and the accumulation of soluble carbohydrates [33]. Low nighttime temperatures can induce chlorosis and damage cellular membranes, thereby reducing water and nutrient uptake [34,35]. Drought reduces cellular turgor and the biosynthesis of primary and secondary metabolites, including naturally occurring antioxidants [30,36].

The conditions exhibited in Figure 2 illustrate how crops are subjected to environmental pressures that disrupt cellular homeostasis and physiological performance. The interaction among multiple stress factors induces alterations in cellular and metabolic processes, which may lead to the development of oxidative stress, a phenomenon that arises in response to redox imbalances triggered by adverse environmental conditions [37]. In lettuce, such conditions may result in reduced leaf growth, impaired root development, loss of cellular turgor, chlorosis, necrosis, and decreased accumulation of bioactive compounds such as vitamins and carotenoids [31,36].

Although biotic and abiotic stresses are conceptually distinct, they share several signaling hubs, including calcium, phytohormones such as abscisic acid (ABA), jasmonic acid (JA), and ethylene (ET), peptide signaling, and ROS. Interactions among these signaling pathways determine the magnitude of the stress response and the allocation of energy and metabolic resources required to activate plant defense mechanisms [38,39].

### 2.1. Abiotic Stress

Abiotic stress encompasses drought, salinity, extreme temperatures (heat and cold), radiation, nutrient imbalances, waterlogging, and exposure to pollutants or heavy metals. In plants, these factors reduce photosynthetic activity, disrupt metabolism, and compromise redox homeostasis through the overproduction of ROS [38,39]. These conditions activate complex signaling networks involving changes in intracellular calcium levels, phytohormones, and ROS, which play a dual role: at controlled levels, ROS function as redox signaling molecules, whereas excessive accumulation leads to oxidative stress and macromolecular damage [40,41].

In lettuce, drought and salinity consistently alter metabolite profiles, including sugars, amino acids, phenolic compounds, and vitamins, while suppressing plant growth, with cultivar-dependent effects. Drought has been associated with relative increases in phenolic compounds and decreases in vitamin C content, whereas salinity reduces the levels of certain amino acids, such as arginine [42]. Moreover, the combination of drought and salinity further exacerbates yield losses and activates antioxidant responses, as evidenced by increased antioxidant activity in assays such as DPPH (2,2-diphenyl-1-picrylhydrazyl) and ABTS [2,2′-azino-bis (3-ethylbenzothiazoline-6-sulfonic acid)], which are widely used to assess antioxidant capacity [43].

Heat stress represents another critical factor in *L. sativa* L., which is particularly sensitive to high temperatures [44,45,46]. Heavy metals are among the most environmentally relevant pollutants, and their accumulation at high concentrations poses a significant environmental risk because of their persistence and capacity to disperse throughout ecosystems [29]. Specifically, Cd is released into these environments through various natural and anthropogenic sources (Figure 3). In addition to disrupting environmental homeostasis, the accumulation of these contaminants promotes stress conditions in living organisms through ROS generation, and other mechanisms associated with physiological and biochemical damage [47]. Consequently, the impact of human activities on environmental quality has become an important area of research, particularly in relation to food security and the sustainability of agricultural production.

### 2.2. Biotic Stress

Biotic stress arises from interactions with pathogens, including fungi, oomycetes, and bacteria, as well as pests and certain weeds. In *Lactuca sativa* L., downy mildew caused by *Bremia lactucae* is one of the most devastating diseases. Recent population analyses have revealed interconnected pathogen lineages associated with *L. sativa* L. and its wild progenitor, *L. serriola*, with host shifts potentially contributing to the rapid breakdown of resistance in commercial cultivars [46].

Another relevant pathogen is *Fusarium oxysporum* f. sp. *lactucae*, the causal agent of *Fusarium* wilt. Under controlled experimental conditions, its impact has been reported to exceed that of water or nutrient deficits, resulting in reduced fresh weight, activation of antioxidant responses, and changes in mineral content. An integrated characterization of spectral traits, pigments, and tissue composition suggests the activation of metabolic defense mechanisms in response to biotic stress [48].

## 3. Oxidative Stress

Abiotic and biotic stressors converge on a common cellular response characterized by disturbances in redox homeostasis and enhanced production of ROS (Figure 4). ROS are highly reactive oxygen-containing molecules, some of which possess unpaired electrons, enabling them to rapidly interact with lipids, proteins, and nucleic acids and thereby alter their structure and function [41,49]. The major ROS include the superoxide anion (O_2_•^−^), hydrogen peroxide (H_2_O_2_), the hydroxyl radical (•OH), and singlet oxygen (^1^O_2_), each exhibiting distinct chemical properties and levels of reactivity (Table 2). Among these, the superoxide anion acts as the main precursor to the formation of other, more harmful ROS.

The production of ROS is a natural byproduct of essential metabolic processes, including photosynthesis in chloroplasts, mitochondrial respiration, and oxidative reactions in peroxisomes. Under normal physiological conditions, ROS production is tightly regulated and maintained at relatively low levels, allowing these molecules to perform regulatory functions in cellular signaling and metabolic adjustment. However, under environmental stress conditions, ROS production increases and may exceed the cell’s antioxidant capacity, ultimately leading to oxidative stress [41]. This redox imbalance can cause membrane lipid peroxidation, protein oxidation (ROS attack amino acids, damaging their structure and affecting their functions), and damage to nucleic acids, compromising the integrity and functionality of the cell. Lipid oxidation alters the fluidity and permeability of cell membranes, while protein modification can affect enzymatic activity and intracellular signaling. Damage to nucleic acids can induce mutations, DNA fragmentation, and affect gene expression, which can lead to premature senescence or programmed cell death [50,51]. The observable physical changes in plants after oxidative stress include wilting, chlorosis, necrosis of leaf tissues, reduced growth, and loss of cell turgor. At the cellular level, these changes reflect membrane alteration, organelle disorganization, and accumulation of oxidized compounds. These effects are visible indicators of oxidative damage and can be measured through physiological and biochemical parameters, such as lipid peroxidation, H_2_O_2_ levels, and antioxidant enzyme activity [41,49]. In the specific case of lettuce, oxidative stress (Figure 4) represents a critical factor that compromises both its development and its nutritional quality.

Several studies have documented that exposure to heavy metals such as cadmium (Cd), lead (Pb), and zinc (Zn), which may be present in treated wastewater used for irrigation, induces excessive ROS accumulation in lettuce leaf tissues, triggering a cascade of adverse physiological and biochemical responses [52]. The most prominent effects include interveinal chlorosis (yellowing of leaf tissue between the veins), marginal necrosis (death of tissue along the leaf margins associated with ROS accumulation in peripheral cells), reduced leaf area, and loss of turgor, resulting in decreased tissue rigidity and firmness (Figure 5). These alterations are primarily associated with lipid peroxidation and the destabilization of cellular membranes.

At the microscopic level, chloroplast disorganization, nuclear condensation, and the accumulation of malondialdehyde (MDA), a widely used biomarker of oxidative damage, have been observed [53,54].

### 3.1. Reactive Oxygen Species Generated During Oxidative Stress

Under physiological conditions, ROS perform essential functions as mediators of cellular signaling, regulators of gene expression, and components of immune defense (Table 2) [55]. However, when ROS production exceeds the antioxidant capacity of the biological system, an imbalance known as oxidative stress occurs, leading to the oxidation of critical biomolecules such as lipids, proteins, and DNA.

ROS can arise from both endogenous and exogenous sources. Major endogenous sources include mitochondrial activity, oxidase enzymes such as NADPH (reduced nicotinamide adenine dinucleotide phosphate), oxidases and xanthine oxidases, and processes occurring within the endoplasmic reticulum. Exogenous sources include environmental factors such as ultraviolet radiation, pollutants, and heavy metals [56]. This dual nature (beneficial at tightly controlled levels but harmful when excessively accumulated) makes ROS key players in cellular physiology and pathology [57,58].

**Table 2:** Biochemical properties, physiological functions, and cellular damage markers of major ROS in plants.

Reactive Oxygen Species	Generation & Biochemical Sources	Cellular Signaling	Cellular Mitigation	Physiological Effects and Damage Markers	Refs.
Superoxide Anion O_2_•^−^	Electron leakage in PSI/PSII. Mitochondrial complexes I/III. Apoplast by NADPH oxidases (RBOHs).	Precursor for the acclimatization of the REDOX response	Dismutation to H_2_O_2_ catalyzed by SOD.	Decrease in photochemical efficiency Membrane damage	[40,59]
Hydrogen Peroxide H_2_O_2_	Dismutation of O_2_•^−^ By-product of photorespiration in peroxisomes	It crosses aquaporins Principal systemic REDOX messenger.	Neutralization via CAT and APX coupled to the Asc-GSH cycle	Alter the structural integrity of the cell wall in the roots	[27,40]
Hydroxyl Radical •OH	Product of the Fenton reaction (H_2_O_2_ + Fe^2+^/Cu^+^) Its formation is amplified by heavy metal stress	Species without a physiological function	It depends on limiting the presence of H_2_O_2_	Immediate biological destruction within the microdomain: severe marginal necrosis, direct lipid cleavage, and DNA strand breaks	[27,40]
Singlet Oxygen ^1^O_2_	Alteration of PSII: anomalous energy transfer from excited chlorophyll	Triggers retrograde signaling from the chloroplast to the cell nucleus Modulates programmed cell death	Physical dissipation of excess light energy Exogenous supply of terpenoids	Lethal oxidation of photosynthetic pigments, tissue turgor, and visible foliar chlorosis	[40,59]

Redox homeostasis emerges from the dynamic balance between the spatial and temporal generation of ROS and their removal by antioxidant systems. This tightly regulated equilibrium allows ROS to function as physiologically useful signaling molecules without exceeding the threshold at which oxidative damage occurs [60,61]. Furthermore, ROS signaling is integrated with phytohormonal pathways, including abscisic acid (ABA) and ethylene, as well as systemic electrical signals, coordinating cell-to-cell and organ-to-organ responses that contribute to plant resilience under combined stress conditions [40].

### 3.2. ROS Generation and Function: An Overview of the Underlying Mechanisms and Their Effects on Plant Systems

Cellular compartments and organelles, including chloroplasts, peroxisomes, mitochondria, and the plasma membrane, also contribute substantially to ROS generation (see details in Table 2). Therefore, a detailed understanding of the mechanisms responsible for ROS production within plant cells is essential for identifying and developing strategies aimed at reducing excessive ROS accumulation and mitigating oxidative damage (Figure 6) [50,62].

#### 3.2.1. Chloroplasts

Chloroplasts convert solar energy into chemical energy in the form of adenosine triphosphate (ATP) and reduced nicotinamide adenine dinucleotide phosphate (NADPH). This energy conversion takes place in the thylakoid membranes, which also represent a major site of ROS generation [63], particularly O_2_•^−^ and ^1^O_2_ [64]. In the presence of light, the oxygen-evolving complex (OEC) of photosystem II (PSII) catalyzes the photolysis of water, resulting in the release of molecular oxygen. However, photosystem I (PSI) is also a major site of ROS generation, where, under conditions of excessive reduction of ferredoxin and limited NADP^+^ availability, molecular oxygen can act as an alternative electron acceptor, resulting in the formation of superoxide (Figure 7) [59].

Under high-light conditions, PSII contributes to ROS formation through processes associated with excitation energy and electron transport. Singlet oxygen (^1^O_2_) is generated through energy transfer from triplet-state chlorophyll to molecular oxygen. Triplet chlorophyll can arise through intersystem crossing from excited singlet chlorophyll within the PSII antenna complexes or through charge recombination of the radical pair within the PSII reaction center. Triplet carbonyl species can also be generated during lipid peroxidation and subsequently transfer energy to molecular oxygen, thereby producing ^1^O_2_ [65].

#### 3.2.2. Mitochondria

In mitochondria, ROS are generated as a result of electron leakage from specific components of the respiratory electron transport chain (ETC). Rather than following their normal electron-transfer pathway, leaked electrons can be transferred directly to molecular oxygen, resulting in the formation of the superoxide anion (O_2_•^−^) (Figure 8) [66].

The main sites of free radical generation are complexes I and III of the ETC. Both complexes release superoxide into the mitochondrial matrix, whereas complex III can also release superoxide into the intermembrane space (IMS) [67].

Mitochondrial ROS generation is normally counteracted by the plant antioxidant system, which comprises a network of enzymes and molecules that neutralize ROS and maintain cellular redox homeostasis. However, when ROS production overwhelms these antioxidant defenses, oxidative stress develops, leading to cellular damage and potentially triggering processes such as necrosis and programmed cell death, a tightly regulated mechanism through which cells activate specific pathways leading to their elimination [68].

#### 3.2.3. Plasma Membrane

Plant membrane-bound nicotinamide adenine dinucleotide phosphate (NADPH) oxidases (NOXs) are transmembrane proteins comprising a membrane-associated respiratory burst oxidase homolog and cytosolic regulatory components. NOXs transfer electrons from intracellular NADPH across the plasma membrane to molecular oxygen in the apoplast, resulting in the production of superoxide (O_2_•^−^). Superoxide can subsequently undergo dismutation to form hydrogen peroxide (H_2_O_2_) [69].

#### 3.2.4. Peroxisomes

Peroxisomes contain two characteristic classes of enzymes, catalase and flavin-dependent oxidases, which are involved in oxidative metabolism and the regulation of hydrogen peroxide levels. Peroxisomal oxidases contribute to H_2_O_2_ generation, whereas catalase plays a major role in its detoxification. The peroxisomal membrane also represents a potential source of ROS, particularly superoxide (O_2_•^−^), due to the presence of an electron transport chain that uses NADH as an electron donor (Figure 9) [70,71].

From seed germination to plant senescence, ROS are dynamically generated and scavenged, allowing plants to regulate their growth and development in response to changing environmental conditions.

However, the effects of ROS on plant growth and development are highly complex because of the spatial and temporal variability of ROS production and removal, as well as the interactions among different ROS within plant tissues [66].

In cellular signaling, ROS are highly versatile molecules because of their distinct physicochemical properties, including specific sites of production, different levels of reactivity, and varying capacities to cross biological membranes. These molecules were initially used by cells to detect potentially harmful levels of atmospheric oxygen and to regulate various metabolic activities; however, they have evolved into potent signaling molecules that regulate a wide range of biological processes in plants, animals, and other eukaryotic organisms [66,72]. In plants, ROS regulate cell differentiation and development, stress signaling, plant interactions, systemic responses, cell death, and cellular redox status.

Understanding the mechanisms of ROS generation and their effects on plant systems is essential for identifying the cellular and physiological processes affected by oxidative imbalance and for developing strategies to improve plants’ tolerance to environmental stress. This knowledge is particularly relevant in lettuce, where environmental stress caused by heavy metal can markedly affect growth, metabolism, and overall plant performance.

## 4. Heavy Metal-Induced Oxidative Stress

Heavy metal stress represents a growing threat to horticultural production due to the persistence and bioaccumulation of these elements and their ability to induce oxidative stress in plant tissues. Both non-essential elements, such as cadmium, lead, mercury, and arsenic, and essential metals, such as copper, zinc, and nickel, can induce redox imbalances when they accumulate above physiological thresholds, thereby impairing photosynthesis, metabolism, and cellular integrity [47,73]. In *Lactuca sativa* L., these effects vary according to the chemical and redox properties of each metal and the mechanisms involved in ROS generation and antioxidant imbalance. These characteristics and their associated physiological effects are summarized in Table 3.

**Table 3:** Chemical properties, ROS induction mechanisms, and physiological effects of heavy metals in *Lactuca sativa* L.

Heavy Metal	Chemical & REDOX Properties	Mechanisms on ROS Induction & Interaction	Physiological Impact in *Lactuca sativa* L.	Refs.
Cadmium (Cd)	Highly mobile and bioavailable in soil/water Not REDOX-active Highly thiol-reactive	Promotes H_2_O_2_ accumulation Displaces essential nutrients Depletes GSH reserves	Reduces root activity Alters the Fv/Fm parameter Induces MDA and proline accumulation	[74,75,76]
Copper (Cu) & Zinc (Zn)	Physiological sufficiency/toxicity threshold Cu is REDOX-active Zn competes for membrane transporters	Cu catalyzes the formation of •OH Zn competitively antagonizes Cd uptakes	Excess Cu reduces biomass and photosynthetic pigments	[47,73,74,77]
Lead (Pb)	Persistent soil pollutant Not REDOX-active Reactive towards thiol groups	Promotes ROS Inhibits antioxidants enzyme activity Displaces essential cofactors	Reduces plant biomass Alters PSII function Triggers lipid peroxidation	[26,73,78]
Mercury (Hg)	Extremely high affinity for thiol groups (-SH)	It sequesters GSH and Asc, affecting cellular antioxidant defense	Increases lipid peroxidation Alters O_2_•^−^ and H_2_O_2_ levels Causes severe structural damage to PSII	[47,73]
Arsenic (As) & Chromium (Cr)	As accumulates alongside Cd and Pb Cr (VI) is highly mobile and reactive	As alters energy metabolism Cr (VI) generates ROS intermediates	Severe genomic damage They disrupt cellular ionic homeostasis The impairing of photosynthetic pigments	[26,47,78,79,80,81]
Nickel (Ni)	Pro-oxidant effects at excessive concentrations	Overloads basal antioxidant defenses Systemic REDOX imbalances	Chromosomal damage Lipid peroxidation Structural deterioration of tissues	[82]

Regardless of their nutritional essentiality, excessive concentrations of heavy metals tend to increase ROS levels (O_2_•^−^, H_2_O_2_, •OH, and ^1^O_2_) and impair antioxidant defense systems, including SOD, CAT, and APX, leading to lipid peroxidation, protein oxidation, DNA damage, and alterations in PSII function [26,47]. In lettuce, these effects are manifested as biomass loss, increased electrolyte leakage, accumulation of H_2_O_2_ and proline, ionic imbalances, and alterations in photosynthetic pigment content (Figure 4) [74,75].

The major routes through which heavy metals enter agricultural production systems include irrigation with contaminated water, the use of phosphate fertilizers, and improperly managed organic amendments, industrial effluents, and atmospheric deposition [74,78]. The following sections summarize the priority metals and metalloids based on their toxicological and agronomic relevance to *Lactuca sativa* L. and other leafy vegetables.

### 4.1. Mechanisms of ROS Production Induced by Heavy Metals in Plants

Individual metals activate distinct but interconnected pathways that can shift physiologically useful redox signaling toward oxidative stress. These mechanisms include direct redox chemistry through Fe- and Cu-mediated Fenton/Haber–Weiss reactions [47,73], disruption of the electron transport chains in chloroplasts and mitochondria [40], and thiol sequestration and displacement of essential cofactors, which impair antioxidant defense systems [79]. The major mechanisms involved in heavy metal-induced ROS production are described below.

1. Direct chemistry: Fenton/Haber–Weiss reactions and redox cycling

The transition metals Cu and Fe catalyze the conversion of H_2_O_2_ into •OH through Fenton chemistry and facilitate redox cycling that promotes O_2_•^−^ generation, thereby amplifying oxidative damage. Although Cd and Pb are not themselves redox-active, their accumulation promotes H_2_O_2_ production and disrupts antioxidant defenses, increasing cellular susceptibility to Cu- and Fe-mediated Fenton reactions [47,73].

2. Disruption of the electron transport chain in chloroplasts and mitochondria

Exposure to heavy metals disrupts chloroplast photochemistry (PSII/PSI) and the mitochondrial respiratory electron transport chain, promoting electron leakage to molecular oxygen and the subsequent accumulation of H_2_O_2_. Damage to PSII also favors the generation of singlet oxygen (^1^O_2_) through energy-transfer reactions (Table 3) [40,47].

3. Thiol sequestration and inhibition of antioxidant defense systems

Many heavy metals exhibit a high affinity for thiol (–SH) groups, thereby depleting glutathione (GSH) and ascorbate (Asc) pools and inhibiting key antioxidant enzymes, including superoxide dismutase (SOD), catalase (CAT), ascorbate peroxidase (APX), and glutathione reductase (GR). These effects impair ROS scavenging capacity and consequently increase oxidative damage [47,73]. In addition, non-essential metals such as Cd and Pb can displace essential metal cofactors, including Zn, Cu, Fe, and Mn, from metalloproteins, particularly those located in chloroplasts, resulting in the loss of enzymatic activity and secondary ROS accumulation [26,79].

Of the heavy metal contaminants, Cd deserves special attention due to its high mobility and superior phytoavailability in agricultural substrates [83]. Unlike essential transition elements, Cd readily crosses root cell membranes via shared divalent cation transporters, facilitating its rapid translocation to the edible part of leafy green vegetables such as *Lactuca sativa* L. [84]. Consequently, understanding its distinctive phytotoxic characteristics and specialized mitigation pathways remains an important goal for crop protection and food safety [85].

### 4.2. Cadmium-Induced Oxidative Stress

When cadmium (Cd) enters the plant, it promotes the generation of ROS, including O_2_•^−^, H_2_O_2_, and •OH, through three major mechanisms: disruption of electron transport in chloroplasts and mitochondria; depletion of thiol-containing compounds, particularly glutathione; and inhibition of antioxidant enzymes such as SOD, CAT, and APX [86,87,88]. This increase in ROS levels results in lipid peroxidation, protein oxidation, DNA damage, and impaired photosynthetic performance [40,87].

In response to Cd exposure, plants bind Cd to chelating compounds, particularly phytochelatins and glutathione, sequester it within vacuoles, and modulate their antioxidant defense systems. However, when Cd exposure is severe or prolonged, oxidative damage may overwhelm these protective mechanisms, ultimately impairing plant growth and crop quality [86,88]. Therefore, identifying and understanding the major sources of Cd contamination in agricultural systems is essential for limiting its entry into crops.

At the root surface, part of the Cd its retained in the cell wall and apoplast, while the rest enters root cells through common divalent cation transporters [89,90,91]. Once inside, cadmium is transported through the xylem toward the leaves or remains stored in root vacuoles, which ultimately determines how much metal reaches the photosynthetic tissue [88,89].

Cd does not directly participate in redox reactions, but it increases ROS formation through indirect pathways that are well characterized in plants. The first route is the interference of Cd with electron transport in mitochondria and chloroplasts, which increases electron leakage toward oxygen and, therefore, the generation of superoxide. This radical is subsequently converted into H_2_O_2_ by the action of SOD [86,87]. The accumulation of H_2_O_2_ reflects the imbalance between production and elimination and is usually accompanied by a decrease in photochemical yield in PSII, an indicator of oxidative damage in chloroplasts [40,87].

Another key pathway is the alteration of the antioxidant system. Cd binds to residues with thiol groups (cysteine) and to glutathione (GSH), which reduces the pool of thiols available to neutralize ROS and limits the activity of enzymes such as SOD, CAT, and APX; in addition, it forces the use of GSH to synthesize phytochelatins, leaving less capacity to eliminate H_2_O_2_ [87,92]. This decrease in antioxidant activity prolongs the half-life of H_2_O_2_ and favors the propagation of damage in membranes, proteins, and DNA, consistent with elevated lipid peroxidation (MDA) profiles under Cd [84,92].

Cd also activates ROS production in the apoplast through NADPH oxidases (RBOH). Exposure to Cd triggers Ca^2+^ signals, hormones (ABA, ethylene, jasmonates), and MAPK cascades that stimulate RBOH, generating pulses of O_2_•^−^ that are converted into H_2_O_2_ and function as signaling waves; these waves, if not buffered, escalate toward oxidative damage [40,84]. This pathway explains rapid increases in ROS outside the energy organelles and their coupling with defense responses and redox adjustment throughout the leaf [40,84].

At the peroxisomal and metabolic level, Cd diverts the flow toward pathways that produce H_2_O_2_, such as photorespiration (glycolate oxidase) and β-oxidation (acyl-CoA oxidase). When the ionic state and enzymatic activity become unbalanced, these pathways gain importance, and the peroxisome accumulates H_2_O_2_, adding to the increase already originating in mitochondria and chloroplasts. This pattern is observed in tissues exposed to Cd with high H_2_O_2_ and proline profiles, consistent with a combined oxidative/osmotic stress state [84,89].

Although Cd is not redox-active, its indirect action predisposes cells to Fenton chemistry when Fe or Cu are available: excess H_2_O_2_ and changes in Fe/Cu availability facilitate the formation of the hydroxyl radical. This step converts signals into very short-range damage, accelerating lipid peroxidation and DNA breaks under Cd exposure [87,88].

The accumulation and persistence of cadmium in agricultural soils represents a critical threat to crop productivity and food security. To mitigate and/or reduce oxidative stress in agricultural crops, areas such as biotechnology and agricultural research have focused on the development of comprehensive mitigation strategies. These strategies are aimed at limiting the bioavailability of cadmium in the soil to curb absorption and at reprogramming the biochemical response to increase antioxidant capacity.

## 5. Strategies for Mitigating Oxidative Stress

Mitigating oxidative stress requires biotechnological strategies that help reduce cellular damage and bolster the endogenous antioxidant defenses of plants. In recent years, several approaches have emerged to successfully restore redox balance through complementary mechanisms, including ROS scavenging, enhancement of antioxidant defenses, osmotic adjustment, modulation of stress signaling, improved delivery of active compounds, and genetic regulation. Priority was given to strategies supported by evidence in *Lactuca sativa* L, while studies in other plants were included when lettuce specific information was limited. The main strategies currently implemented are described below [27,93,94].

### 5.1. Exogenous Application of Antioxidants

The exogenous application of antioxidants represents an agronomic intervention strategy that has proven effective in mitigating oxidative stress in plants exposed to salinity, heavy metals, drought, and other abiotic stresses. Foliar spraying or priming with antioxidant compounds has been shown to restore redox homeostasis, modulate osmoprotective pathways, and stimulate the biosynthesis of secondary metabolites involved in plant defense. Thus, exogenous treatments can act both as direct ROS-scavenging or chelating agents and as modulators of plant metabolic responses [95]. In the case of ascorbic acid (AsA), extensive experimental evidence indicates that foliar applications improve morphological parameters, enhance antioxidant enzyme activity, and increase osmoregulatory capacity, resulting in improved growth and yield under saline conditions, as demonstrated in crops such as barley and common bean. These findings support the role of AsA as an effective modulator of the AsA–GSH cycle and cellular redox homeostasis [96].

Glutathione (GSH), a central component of the cellular redox system, has also been applied exogenously and has shown protective effects against Cd toxicity and salinity. Experimental studies indicate that foliar GSH application not only increases endogenous GSH concentrations but can also reduce metal translocation, downregulate transporters involved in Cd uptake, and improve the GSH/GSSG ratio, an important indicator of cellular redox status. Evidence from studies in wheat and potato therefore supports the use of exogenous GSH to mitigate heavy metal stress and associated lipid peroxidation [97,98,99]. In parallel, exogenous melatonin has emerged as a multifunctional signaling molecule capable of regulating antioxidant pathways, interacting with phytohormonal networks, and improving photosynthetic performance and membrane integrity. Several recent studies have described its effectiveness against salinity and drought in vegetables and cereal crops, with beneficial effects on both antioxidant enzyme activity and the expression of stress-responsive genes.

Osmoprotectants such as glycine betaine (GB) can also exert functional antioxidant effects. Foliar application or priming with GB enhances osmotic adjustment, protects the photosynthetic machinery, and is associated with increased total antioxidant capacity and enhanced activity of antioxidant enzymes, including SOD, CAT, and peroxidase (POD). Evidence from maize, squash, and other plant species, including recent experimental and meta-analytical studies, supports the potential of GB as a practical and readily applicable strategy under field conditions. Similarly, macroalgal extracts, such as those obtained from *Ulva lactuca*, and microalgal concentrates have demonstrated biostimulant effects by combining the direct supply of carotenoids, vitamins, and phenolic compounds with the induction of endogenous antioxidant pathways. Previous studies and reviews have reported improvements in plant growth, phenolic content, and total antioxidant activity under saline stress following foliar application of algal extracts, supporting their use as natural agents with both nutritional and signaling functions in the mitigation of oxidative stress [100,101].

Regarding carotenoids, studies such as [102], which investigated exogenous β-carotene and *Ulva* extract in wheat, showed that foliar β-carotene application increased the concentrations of compatible osmolytes in shoots, including proline and glycine betaine, while also increasing antioxidant metabolites such as AsA, GSH, and total phenolic compounds. These effects strengthen both direct chemical defenses against ROS and metabolic reprogramming associated with enhanced NaCl tolerance. This study illustrates how an exogenously applied carotenoid can exert a dual function: physicochemical protection against ROS and metabolic signaling that enhances antioxidant pathways and secondary metabolite biosynthesis.

Chitosan, a marine-derived biopolymer, and other biopolymers have also been investigated for their ability to induce plant defense responses. Foliar application of chitosan increases the accumulation of phenolic compounds and flavonoids and enhances antioxidant enzyme activity, providing protection against drought and salinity. Moreover, owing to its physicochemical properties, chitosan can serve as a protective matrix for other compounds in co-application strategies, creating opportunities for combined formulations that extend the foliar persistence of antioxidants susceptible to photodegradation [103].

From a mechanistic perspective, available evidence indicates that the exogenous application of antioxidants operates through three interconnected mechanisms: (i) direct ROS scavenging and prevention of lipid peroxidation; (ii) restoration and enhancement of non-enzymatic antioxidant systems, including the AsA–GSH cycle, phenolic compounds, and tocopherols, as well as enzymatic defenses such as SOD, CAT, and APX; and (iii) regulation of metabolic and signaling pathways that induce the synthesis of osmolytes and phenolic compounds and modify the expression of stress-responsive genes. Together, these mechanisms explain why exogenous antioxidant treatments consistently improve physiological parameters, including chlorophyll content, maintenance of plant water status, and crop yield, across a wide range of plant species and stress conditions [95].

Nevertheless, exogenous applications present several practical limitations and challenges highlighted in the literature. These include the chemical instability of many antioxidants, particularly carotenoids and AsA, when exposed to light and temperature; limited foliar penetration; the need to determine optimal doses and application frequencies; and potential interactions with crop physiology and phenological stage. Therefore, experimental protocols (including concentration, vehicle formulation, application volume per plant, and number of applications) must be standardized to facilitate comparisons among studies and generate reproducible agronomic recommendations [95].

Synergistic combinations of antioxidants with other approaches, including biostimulants, plant growth regulators, encapsulation systems, and nanoformulations, may further enhance efficacy and overcome limitations related to stability and bioavailability. In this context, combining exogenous antioxidants with controlled-release delivery systems, such as nanoparticles or encapsulation within inorganic matrices or biopolymers, represents a promising strategy to increase foliar persistence, provide sustained release, and optimize antioxidant bioavailability at the cellular level.

### 5.2. Use of Biostimulants

The use of biostimulants has become established as an environmentally friendly biotechnological strategy capable of modulating key physiological processes, including antioxidant defense, hormonal signaling, and osmotic adjustment, thereby improving plant performance under abiotic stress, including stress induced by heavy metals. According to the international regulatory framework for fertilizer products, biostimulants are divided into two main categories: non-microbial and microbial [104,105,106].

Among non-microbial biostimulants, the seaweed extracts, particularly those derived from *Ascophyllum nodosum*, *Ecklonia maxima*, and *Ulva* spp., contain polysaccharides, polyphenols, and other bioactive fractions that activate stress-tolerance pathways and contribute to the maintenance of redox homeostasis. These effects have been attributed primarily to the ability of seaweed extracts to modulate gene expression and antioxidant responses rather than simply to the exogenous supply of phytohormones [107]. In lettuce subjected to salinity stress, the application of seaweed-derived bioproducts has been associated with improvements in photosynthetic pigment content and ionic balance, together with significant reductions in H_2_O_2_ and malondialdehyde (MDA) levels, indicating mitigation of oxidative stress [45]. A recent study demonstrated that the application of *Ecklonia maxima* extracts increased leaf iron content and improved physiological biomarkers, establishing a potential link between biostimulation and the nutritional quality of crops for human consumption [108]. However, variability in seaweed species, extraction methods, and product composition can result in distinct physiological responses, highlighting the need for detailed compositional characterization and optimization of application doses and timing [109].

Polyphenols, including flavonoids and phenolic acids, play important roles in ROS detoxification and membrane protection. In lettuce, moderate salinity management can increase the accumulation of compounds such as caffeic acid, chlorogenic acid, quercetin, and rutin without compromising crop quality, thereby strengthening endogenous antioxidant pools [45]. More broadly, activation of the phenylpropanoid pathway and subsequent polyphenol accumulation have been recognized as key mechanisms underlying tolerance to abiotic stress [110]. Nevertheless, a potential limitation of exogenous polyphenol application is the excessive suppression of ROS signaling when antioxidants are administered at high doses, because controlled hormetic ROS signals are required for appropriate stress acclimation and adaptation [40].

Amino acids and protein hydrolysates are also used as biostimulants. These compounds can act as osmoprotectants, precursors of phytohormones, and chelators of micronutrients, thereby promoting antioxidant enzyme activity and proline accumulation under adverse environmental conditions. In lettuce, combining microbial inoculants with protein hydrolysates has been shown to improve crop yield and root architecture while enhancing antioxidant status [111]. However, variability in the composition of protein hydrolysates and cultivar-specific responses requires these strategies to be optimized according to plant genotype [112]. Humic and fulvic substances can also enhance nutrient-use efficiency, photosynthetic activity, and antioxidant capacity. In crops such as lettuce, these substances have been associated with reduced ROS and MDA levels, improved electron transport, and enhanced tolerance to cadmium. These protective effects have been attributed, at least in part, to increased antioxidant capacity and enhanced thiol biosynthesis [113,114].

Microbial biostimulants, particularly plant growth-promoting rhizobacteria (PGPR) and arbuscular mycorrhizal fungi (AMF), play a critical role in alleviating heavy metal toxicity. Under cadmium stress, these microorganisms act as both biophysical and biochemical barriers within the rhizosphere. PGPR strains, such as those from the *Bacillus* and *Pseudomonas* genera, secrete exopolysaccharides and siderophores capable of trapping and immobilizing metal cations directly in the soil. Additionally, they produce 1-aminocyclopropane-1-carboxylate (ACC) deaminase, an enzyme that breaks down the immediate precursor of ethylene, thereby delaying cellular senescence and rescuing root growth [115]. Inoculation with AMF drives glomalin secretion and enhances metal retention within the fungal hyphal cell walls, effectively restricting cadmium translocation to the photosynthetic tissues [116]. At the molecular level, this microbial symbiosis triggers transcriptional reprogramming in the host plant. By inducing systemic resistance, it upregulates endogenous antioxidant enzymes while reducing oxidative stress like MDA and H_2_O_2_ [117]. Beyond their individual applications, the co-inoculation of PGPR and AMF provides striking synergistic benefits for alleviating heavy metal stress. This combined root colonization essentially reshapes the rhizosphere microbiome; it not only amplifies metal biosorption but also strengthens the cellular signaling required to sustain REDOX homeostasis [118]. A further key mechanism involves the modulation of phytohormonal crosstalk alongside the synthesis of specialized metabolites. This interplay directly boosts the pool of critical non-enzymatic antioxidants, particularly glutathione. Such an increase is vital for phytochelatin biosynthesis. These oligopeptides act by chelating intracellular cadmium ions and sequestering them safely within the vacuoles, effectively shielding the cytosol from oxidative damage [116,118].

### 5.3. Seed Priming and Nanopriming Techniques

Seed priming is understood as a hydro-physiological pretreatment that activates early metabolic pathways and defense systems in the seed without allowing radicle protrusion; the purpose is to accelerate and synchronize germination, strengthen seedling establishment, and increase tolerance to abiotic stress in horticulture. In lettuce crops, there is evidence confirming that priming improves root growth, gas exchange, and the accumulation of osmolytes (such as proline), while reducing electrolyte leakage and membrane damage under exposure to salt stress [119]. At the conceptual level, priming is understood to establish a physiological memory that facilitates faster and more efficient responses to a subsequent challenge; this memory is associated with transcriptional reprogramming, adjustment of hormonal pathways, repair of molecular damage, and modulation of ROS homeostasis and enzymatic antioxidant systems (SOD, CAT, APX, GR), which together preserve photosynthetic pigments (SPAD) and photosystem II efficiency when seedlings face stress [120,121].

Hydropriming is distinguished by its low cost, reproducibility, and broad applicability in horticultural crops; some reviews recommend this method as the first option in systems seeking uniformity and rapid emergence, provided that oxygenation is ensured during imbibition and careful drying is performed to prevent radicle protrusion and thus preserve storage capacity and the physiological quality of the seed lot [120,122]. Osmopriming/halopriming, which uses solutions with reduced water potential (salts such as KNO_3_ or CaCl_2_) to modulate imbibition kinetics and pre-acclimate the seed to osmotic/ionic stress, has shown efficacy in lettuce exposed to salt stress, with early physiological improvements translated into greater vigor and membrane stability during the first days; these effects have been documented under controlled conditions and with integrated measurements of germination, root growth, gas exchange, and biomarkers such as proline and MDA [119,122]. Chemopriming with compatible molecules, including proline (20 mM), has demonstrated benefits under heavy metal stress (cadmium) in *Lactuca sativa* L. Priming with proline increased germination, chlorophyll content, and improved Zn/Fe uptake in roots, suggesting a combination of osmotic buffering and antioxidant activation capable of sustaining the photosynthetic apparatus during critical stages [123].

Nanopriming emerges as an evolution of conventional priming by incorporating nanoparticles (NPs) and bio-based nanocarriers to couple metabolic signals (α-amylase, reserves) and reinforce the antioxidant network with more precise control of dose and delivery kinetics. Recent studies propose ZnO and SiO2 NPs because of their cost–benefit ratio and their relatively stable chemistry in priming systems; at this scale, nanopriming buffers ROS peaks and enhances the activity of the enzymes SOD, CAT, APX, and GR, increases proline and sugars, and improves root architecture, with subsequent gains in membrane integrity and photosynthetic efficiency when plants face heat, drought, or salinity [124,125].

Some representative examples of nanopriming applications are presented in Table 4.

### 5.4. Application of Particles, Nanoparticles, and Encapsulated Systems

The application of nanoparticles and encapsulated systems in agriculture involves the use of nanoscale carriers (1–100 nm) to improve the delivery of nutrients, biostimulants, and active compounds by enhancing their stability, bioavailability, and controlled release. These systems can enter plants through seeds, roots, or foliar tissues and subsequently be translocated through plant tissues via apoplastic and symplastic pathways, resulting in greater physiological efficiency and reduced losses through leaching or degradation [129,130]. The nanoparticles most used in horticulture include metal oxides such as ZnO and TiO_2_, amorphous silica (SiO_2_), calcium phosphates such as hydroxyapatite, nanoclays, and zeolites, as well as biodegradable polymeric carriers such as chitosan, alginate, and starch. These materials are generally selected because of their high surface area and ability to interact with biological surfaces [130,131].

In *Lactuca sativa* L., recent evidence indicates that foliar application of nanoparticles can improve stress tolerance and crop quality. For example, under cadmium toxicity, foliar application of SiO_2_ (50–100 mg L^−1^), ZnO (50–100 mg L^−1^), and TiO_2_ (20–60 mg L^−1^) increased biomass, improved root architecture and photosynthetic efficiency, reduced ROS and MDA accumulation, and enhanced the activity of antioxidant enzymes such as SOD, POD, CAT, and APX. These treatments also reduced Cd concentrations in roots and leaves by approximately 10–72% [132]. However, other studies have highlighted potential phytotoxic effects. TiO_2_ applied at concentrations above 50 mg L^−1^ induced leaf necrosis and chlorosis and reduced biomass when applied directly to seeds, emphasizing the need to establish safe concentration ranges and to distinguish between foliar and seed-based application methods [133].

Beyond metal oxides, nanofertilizers based on nanoclays, zeolites, hydroxyapatite, and mesoporous silica have been shown to improve nutrient-use efficiency and yield, thanks to their capacity for gradual and targeted release. Recent reviews highlight that these systems reduce application frequency and losses through leaching, although gaps persist between results obtained under controlled conditions and their performance in the field, requiring standardized protocols and multi-site validation before their widespread adoption [131,134]. In the same sense, the nanoencapsulation of agricultural inputs using biodegradable polymers such as chitosan and alginate offers additional advantages: protection against UV radiation, moisture, and oxidation, and controlled release under specific stimuli (pH, temperature, enzymes), which prolongs the activity of the compound and reduces environmental impact [135,136].

In particular, chitosan has become established as a versatile carrier due to its biocompatibility, antimicrobial properties, and chelating capacity. In lettuce, chitosan nanoparticles applied as a biostimulant have increased yield by approximately 25 %, improved the content of bioactive compounds, and increased catalase activity at low doses (0.05–0.1 mg mL^−1^), which confirms their potential as a safe and effective nanoformulation [137]. In general level, chitosan encapsulates for fertilizers and biostimulants have been shown to reduce leaching and improve use efficiency, although the literature warns of the need to evaluate ecotoxicity and establish clear regulatory frameworks to guarantee food safety [138,139].

A critical aspect of the application of nanoparticles and encapsulated systems is safety. Some studies on foliar exposure to nanoplastics in lettuce have confirmed absorption, translocation, and trophic transfer in food chains, in addition to metabolic alterations in energy and antioxidant pathways, which reinforces the importance of characterizing particle size, charge, and concentration and of implementing quantitative methods to assess risks [140,141]. In this context, it is of utmost importance to understand the response mechanisms of plants to different types of stress, from visible changes such as growth, development, and physiology to the modifications that occur at the cellular level. In this way, the effects of the applied strategies can be interpreted in an integrated manner, and thus more exact standards for safe and efficient use can be established.

### 5.5. Genome Editing and Genetic Engineering

Genetic engineering and genome editing, particularly the CRISPR/Cas9 system, have emerged as innovative strategies to enhance the biosynthesis of antioxidant compounds and mitigate oxidative stress. Biofortification demonstrated its agronomic and nutritional viability with the development of “Golden Rice”, which was genetically engineered to accumulate β-carotene in the endosperm as a global strategy against vitamin A deficiency [142]. Recently, this same concept has been successfully translated to *Lactuca sativa* L. through the creation of “Golden Lettuce”. Morelli et al. managed to increase β-Carotene content up to 30–fold in lettuce leaves by redesigning the biosynthetic pathway and directing storage toward plastoglobules. This carotenoid enhancement not only raises the nutritional quality of the crop but also reinforces non-enzymatic defenses against ROS without sacrificing photosynthetic efficiency [143].

CRISPR/Cas9 technology is revolutionizing the modulation of redox responses in agricultural crops, highlighting its use to inactivate negative regulators and redirect the accumulation of protective metabolites. However, the success of the biotechnological interventions relies on the precise identification of genetic targets. For Instance, the compressive characterization of the polyphenol oxidase gene family and its upregulation during wound stress provides the essential transcriptomic framework needed to design CRISPR/Cas9 strategies aimed at mitigating oxidative browning and reducing enzymatic damage in plant tissues [144]. This technology also allows the modification of ion transporters and signaling pathways that govern the antioxidant machinery, enabling control over cellular homeostasis under toxic metal exposure [145]. These biotechnological approaches enable the design of resilient crop varieties with robust antioxidant profiles capable of tolerating various forms of stress.

## 6. Future Perspectives

Scientific evidence indicates that oxidative stress management will shift from isolated solutions to complementary combinations. In *Lactuca sativa* L., the best performance is achieved when physiological (seed priming and nanopriming), biochemical (exogenous antioxidants), nutritional (micronutrients and humic/fulvic substances), and nanotechnological (carriers, nanoencapsulated systems, genome editing, and genetic engineering) strategies are combined to maintain redox balance without suppressing the signals necessary for adaptation [38,40,41]. Previous results show that plant and microbial biostimulants, as well as humic acids, improve pigments, ionic balance, and antioxidant activity under salinity and metal stress [45,113,114,146]. At the same time, nanoparticles and nano/microstructured systems can increase the stability and efficacy of active compounds, with benefits in growth, photosynthesis, and ROS reduction, provided that doses and application times are adjusted [119,122,130,131,132].

In the future, it will be of great importance to validate the efficiency of these strategies under different types of stress or a combination thereof (salinity–heat, metals–drought), improve the bioavailability and release of active compounds, to ensure sustainability and safety. It should be noted that nano/microstructured carriers help protect sensitive molecules (ascorbic acid, carotenoids) and deliver them gradually; due to their high surface area and exchange capacity, porous supports such as zeolites are emerging as useful platforms within the group of nanofertilizers and slow-release systems [130,131,134,135,139].

The challenge is to refine safe windows for the size, charge, and concentration of nanocarriers to avoid phytotoxicity and variability among cultivars; evidence in lettuce shows benefits but also adverse effects if doses are exceeded or salts are combined inadequately [127,132,133]. Likewise, difficulties remain to be overcome, such as the solubility, dispersion, and stability of encapsulated bioactive compounds, since these factors directly influence bioavailability, adsorption, transport, and efficacy within plant tissues. Similarly, the environmental fate and trophic transfer of carriers should be monitored, adjusting evaluation and traceability protocols for leafy vegetables [140,141]. Overall, this scientific review offers a more precise and robust pathway to mitigate oxidative stress and sustain crop performance.

## 7. Conclusions

Based on the present review, oxidative stress in *Lactuca sativa L*. is a common axis of damage under salinity, drought, extreme temperatures, and exposure to heavy metals, due to the increase in ROS and the disruption of antioxidant systems. Scientific evidence indicates that the mitigation of oxidative stress may be more effective if strategies are combined: exogenous antioxidants, biostimulants, nutritional management, seed priming/nanopriming, nano/microstructured carriers that improve the stability and release of active compounds, and genome editing and genetic engineering.

For their widespread adoption in the field, greater emphasis should be placed on standardizing protocols (dose, vehicle, and phenological stage), validating them under combined stress, and monitoring the safety and traceability of carriers to avoid phytotoxicity, accumulation, and trophic transfer. Comparative trials in different cultivars and systems (open field, greenhouse, hydroponics), together with agronomic and quality metrics, will allow physiological benefits to be translated into yield and commercial value. In summary, integrated and precision management represents the most consistent pathway to sustain redox balance and lettuce productivity under increasingly complex stress scenarios.

## Figures and Tables

**Figure 1:**
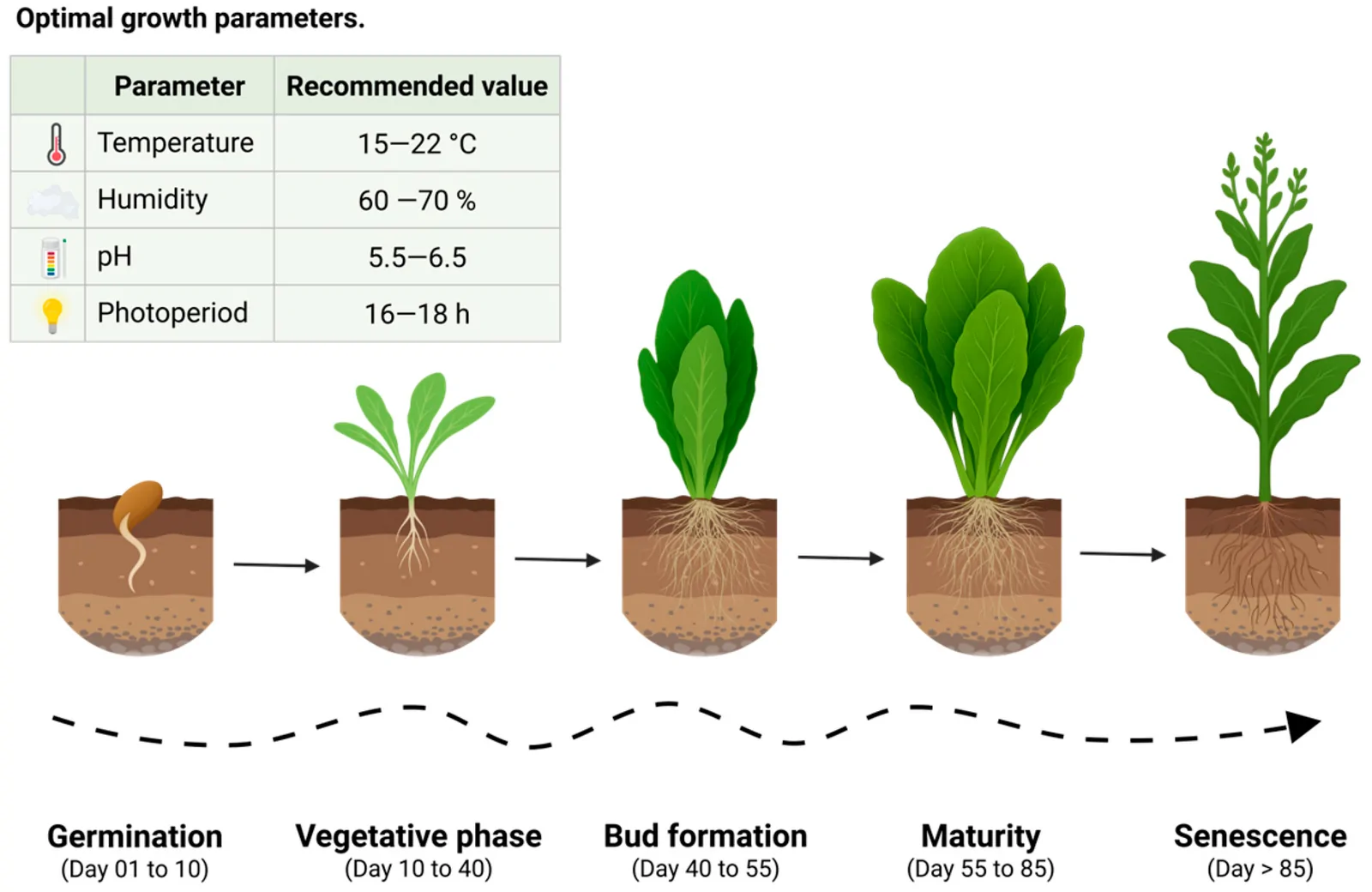
Developmental stages of lettuce (*Lactuca sativa* L.) from germination to senescence and recommended environmental parameters for optimal growth. Source. Based on [18].

**Figure 2:**
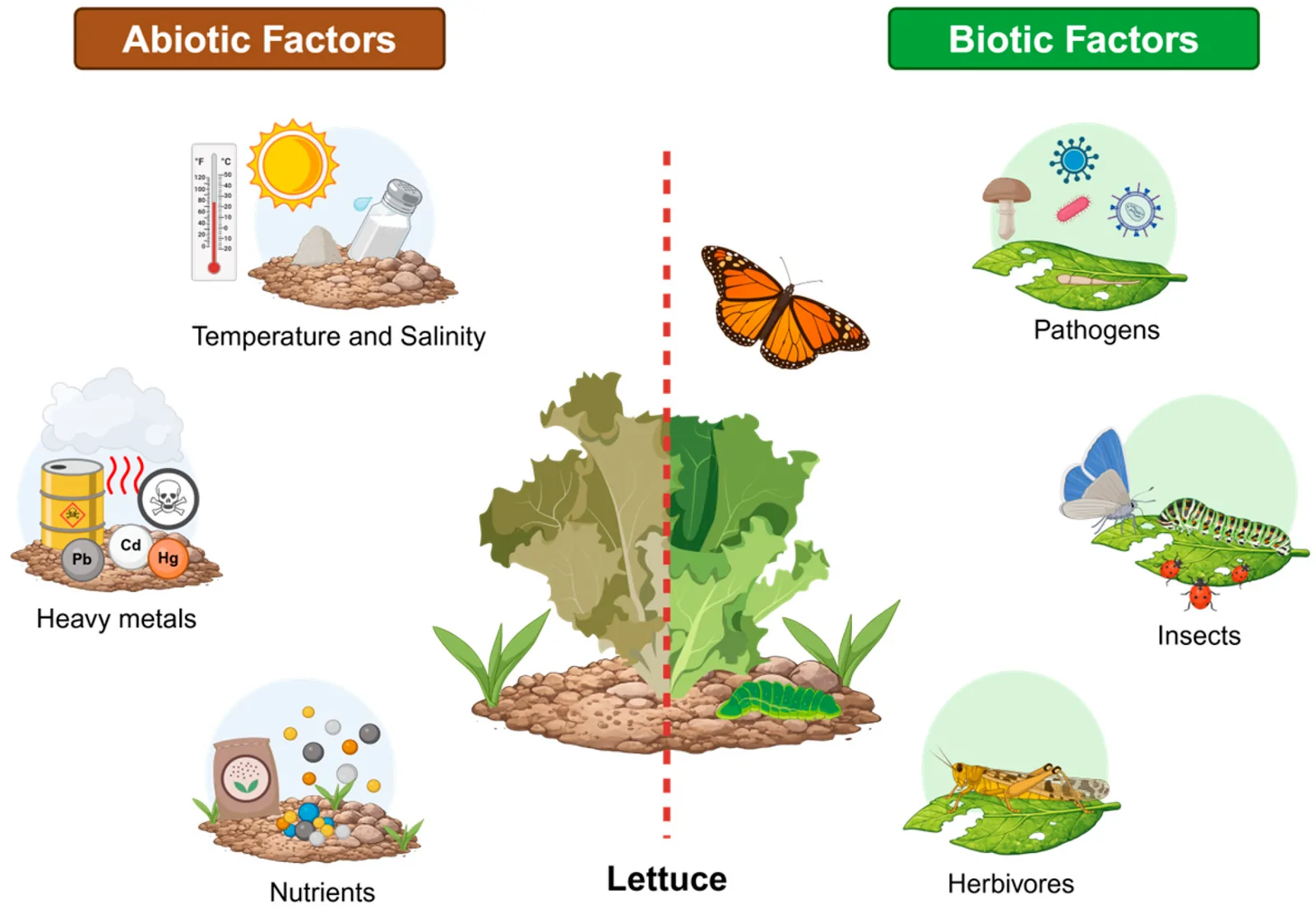
Primary biotic and abiotic environmental stressors affect physiological homeostasis in agricultural crops.

**Figure 3:**
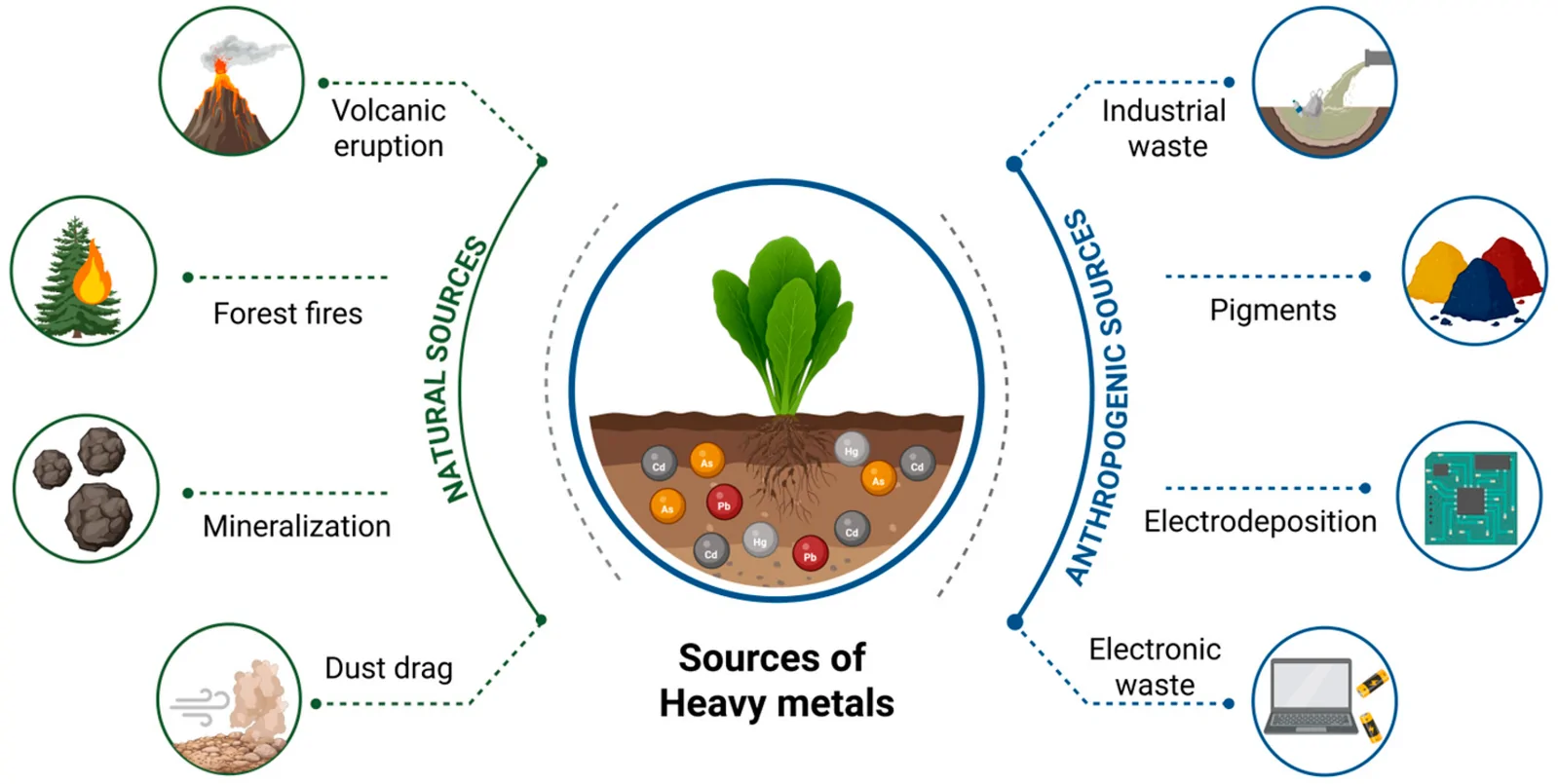
Natural and anthropogenic sources of heavy metal contamination and their potential entry into the soil–plant system.

**Figure 4:**
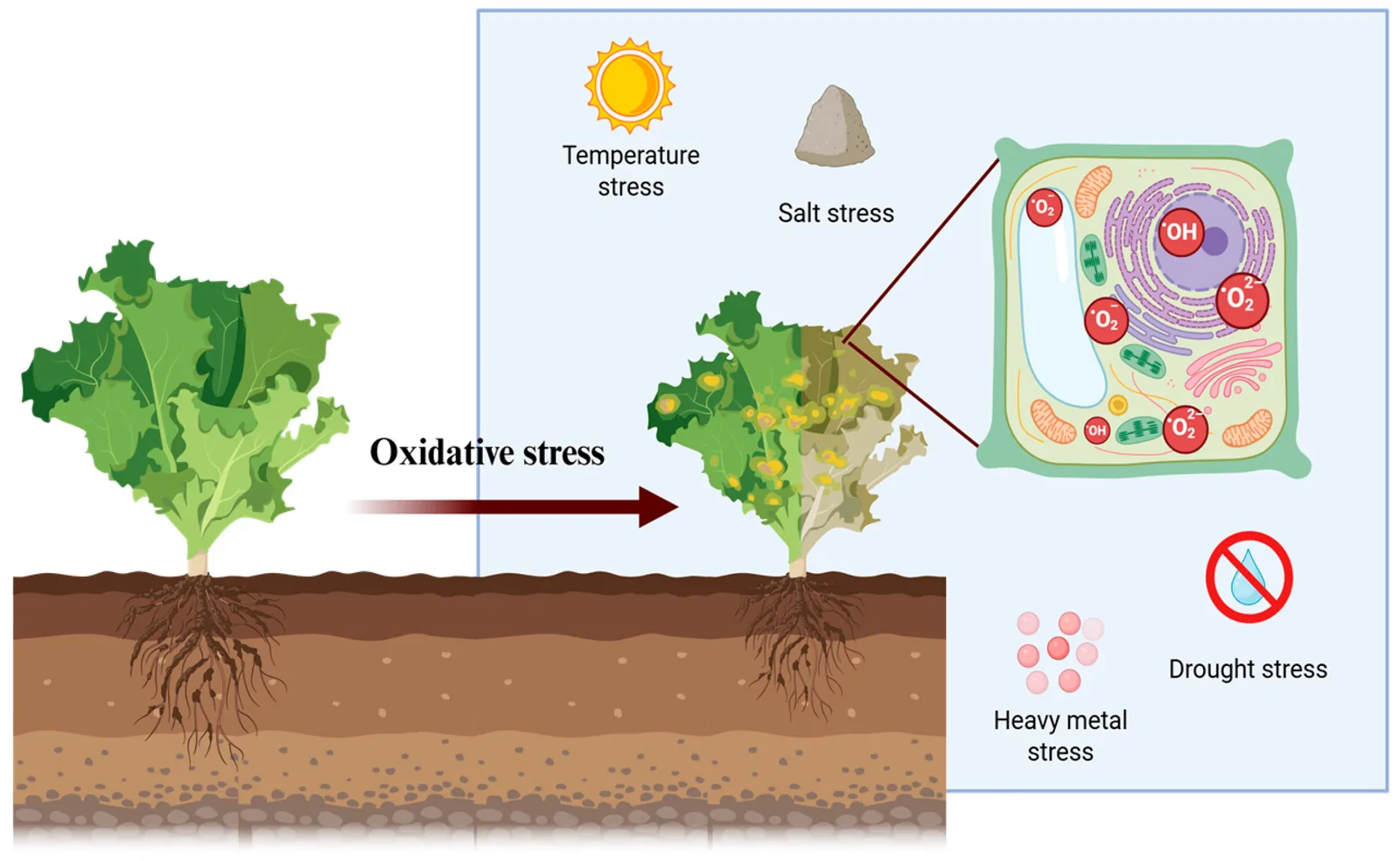
Oxidative stress and subcellular ROS accumulation in response to adverse environmental conditions.

**Figure 5:**
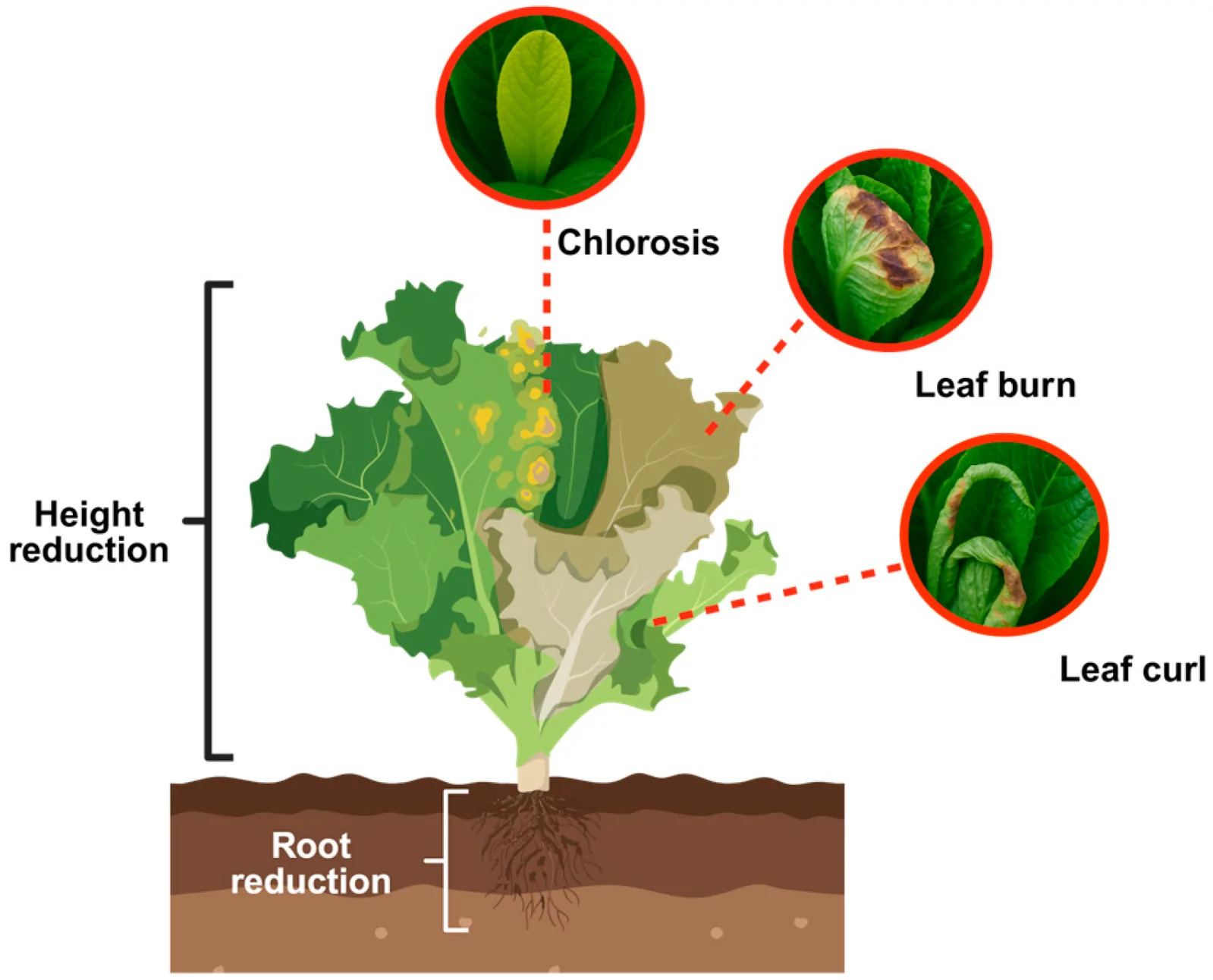
Macroscopic manifestations of oxidative stress in lettuce: chlorosis, necrosis, and morphological alterations.

**Figure 6:**
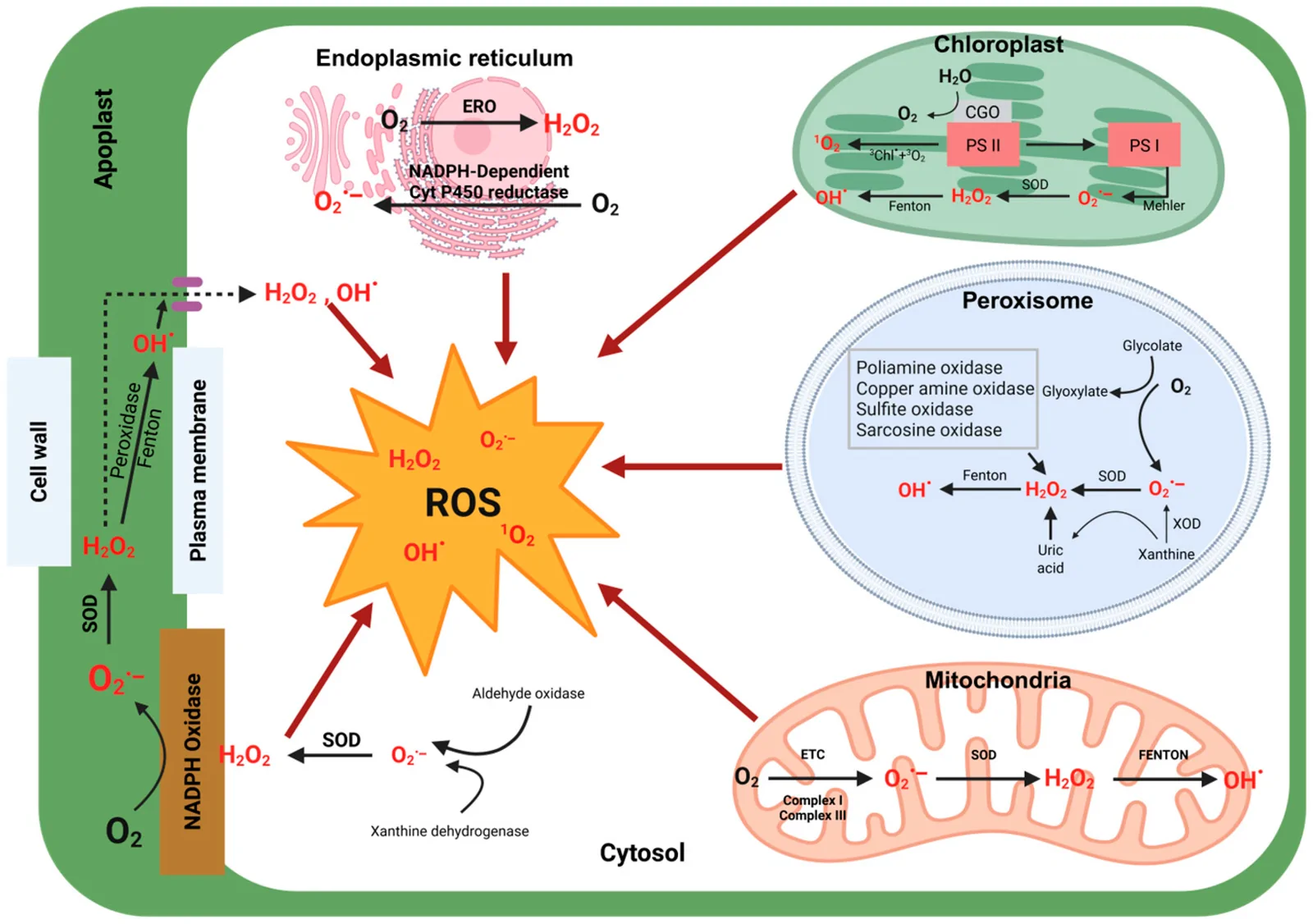
Processes involved in ROS generation in plant cells. ROS (Reactive oxygen species), H_2_O_2_ (hydrogen peroxide), O_2_•^−^ (superoxide anion); ^1^O_2_ (singlet oxygen); •OH (hydroxyl radical); SOD (superoxide dismutase); XOD (xanthine oxidase); ETC (electron transport chain); PSI (photosystem I); PSII (photosystem II); NADPH (nicotinamide adenine dinucleotide phosphate). Source. Modified from [27].

**Figure 7:**
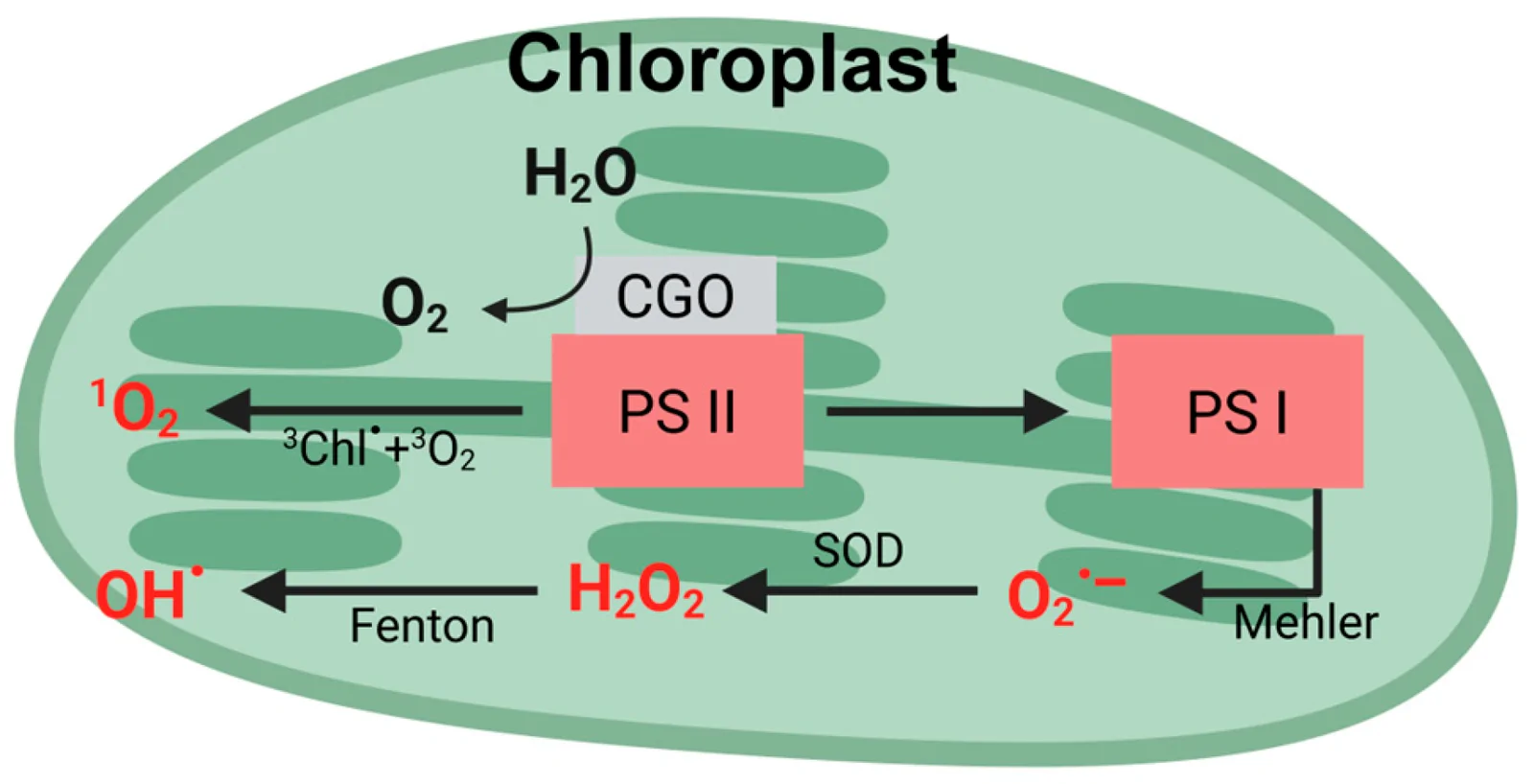
Electron transport chain disruption and ROS production sites within the chloroplast photosystems (PSI and PSII).

**Figure 8:**
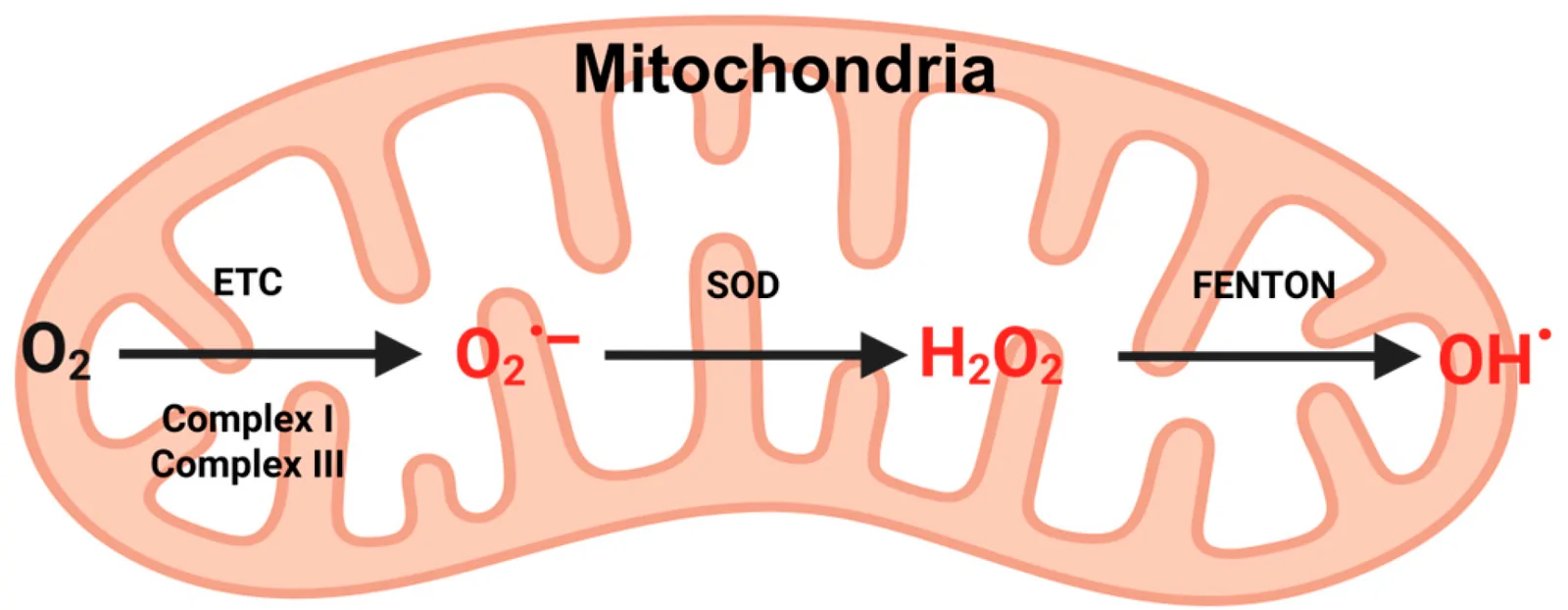
Mitochondrial pathways contribute to intracellular superoxide and hydrogen peroxide accumulation.

**Figure 9:**
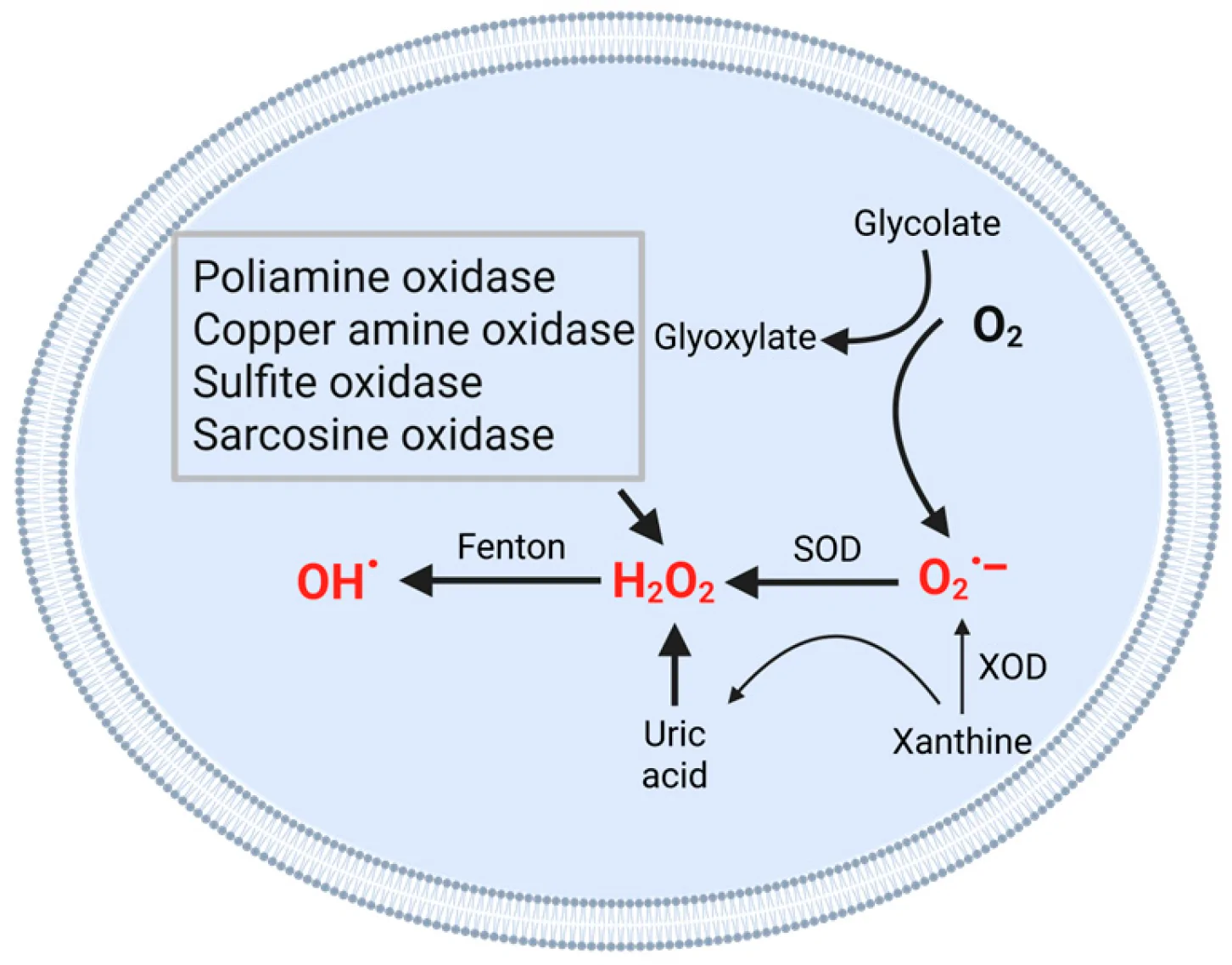
Peroxisomal oxidative metabolism and hydrogen peroxide regulation during cellular stress.

**Table 1:** Nutritional profile and primary/secondary metabolite composition of *Lactuca sativa* L.

	Category	Components
Primary metabolites	Macronutrients	Carbohydrates, proteins, lipids (omega-3 fatty acids, linolenic acid), structural polysaccharides.
	Amino acids	Alanine, arginine, aspartic acid, cysteine, glutamic acid, glycine, histidine, isoleucine, leucine, lysine, methionine, phenylalanine, proline, serine, threonine, tryptophan, tyrosine.
	Organic acids	Lactic acid.
	Structural components	Ash, water.
	Vitamins	B-complex vitamins, biotin, choline, folate, pantothenic acid, vitamin C (ascorbic acid), vitamin A, vitamin D, vitamin E.
	Minerals	Boron, calcium, chloride, chromium, copper, iodine, iron, magnesium, manganese, molybdenum, phosphorus, potassium, selenium, sodium, zinc.
Secondary metabolites	Phenolic compounds	Total phenols, tannins, flavonoids, flavonols, quercetin, caffeic acid.
	Terpenoids	Carotenoids, lactucin, lactucopicrin
	Other compounds	Plant steroids, glycosides, plant gums.

Source. Modified from [23].

**Table 4:** Effects of nanopriming on physiological and biochemical responses in lettuce.

Material	Conditions/Stress	Physiological and Biochemical Responses	Observations	References
ZnO (NPs)	Nanopriming (25–50 mg/L), under basal conditions	Increased plant length and internal Zn content	Confirmed NP uptake and enhanced antioxidant activity	[126]
SiO_2_	Hydroponic conditions, CaCl_2_ stress (100 mg/L de SiO_2_)	Increased biomass and non-enzymatic antioxidant capacity	Effective mitigation of CaCl_2_-induced stress	[127]
ZnO	Hydroponic conditions, CaCl_2_ stress and combined NaCl + CaCl_2_ (100 mg/L de ZnO)	Antagonistic interaction with CaCl_2_, exacerbation of cellular damage under combined salt stress	Evidence that effectiveness depends on the interaction between the NP and the stress-inducing ion	[127]
ZnO + SiO_2_ in a chitosan matrix	Seed priming, mannitol-induced water stress	Substantial increases in proline, total phenolic content, and antioxidant capacity	Outperformed conventional hydropriming	[128]

## Data Availability

No new data were created or analyzed in this study. Data sharing is not applicable to this article.
